# Giant Cell Reparative Granuloma in the Thumb Distal Phalanx: A Case Report

**DOI:** 10.7759/cureus.43564

**Published:** 2023-08-16

**Authors:** Varun Arvind, Matthew W Konigsberg, Robert J Strauch

**Affiliations:** 1 Orthopedic Surgery, Columbia University College of Physicians and Surgeons, New York, USA; 2 Orthopedic Surgery, Beth Israel Deaconess Medical Center, Harvard Medical School, Boston, USA

**Keywords:** lytic, giant cell tumor, benign lesion, thumb, giant cell reparative granulomas

## Abstract

Giant cell reparative granulomas (GCRG) often affect the bones of the hands and the feet. Treatment of this lesion depends on the exact location and amount of localized bony destruction. Ours is the first case report to discuss the nuances of treating this lesion in the thumb distal phalanx. A 19-year-old male presented with lytic, destructive expansion of his left thumb distal phalanx; imaging was suggestive of an aneurysmal bone cyst. Open biopsy was interpreted as giant cell reparative granuloma. Curettage and bone grafting resulted in complete healing of the distal phalanx with an excellent range of motion and interphalangeal joint stability. GCRG is a rare, benign entity typically presenting as a lytic bone lesion. Despite the initial massive bony destruction, this lesion nevertheless healed with curettage and bone grafting with maintained flexor pollicis longus and extensor pollicis longus function, permitting excellent active motion postoperatively.

## Introduction

Giant cell reparative granuloma (CGRG) is a non-neoplastic, intraosseous reactive bone disease characterized by cellular fibrous tissue containing multiple areas of hemorrhage, multinucleated giant cells, and occasionally woven bone trabeculae [1-6]. CGRC is classified among other fibroosseus lesions of bone including ossifying fibroma, fibrous dysplasia, osteoclastoma, brown tumor, and the aneurysmal bone cyst with overlapping radiographic and histologic features. Therefore, consideration of clinical, radiographic, and histopathology is essential for accurate diagnosis and appropriate treatment of CGRC.

The cause of CGRG remains unknown but may be associated with local trauma, dysregulated repair processes, developmental disturbances, or inflammation [1-3]. The condition primarily affects children and young adults, with the majority of cases occurring before the age of 30 [1]. First characterized by Jaffe in the mandible, CGRC shows a predilection for the craniofacial bones and is rarely seen in bones of the hand or feet [2]. CGRG can be categorized into aggressive and nonaggressive types based on clinical and radiographic features, with the aggressive form being more common in young adults with high recurrence rates of up to 72% [4,6].

Treatment consists of aggressive curettage followed by bony reconstruction, or excision. We describe the case of an adolescent male with a GCRG of his thumb distal phalanx with minimal remaining bone, who had an excellent outcome after curettage and cancellous bone grafting.

## Case presentation

History and examination

The patient was a 19-year-old healthy male who presented with two months of atraumatic swelling and one week of pain in his non-dominant thumb distal phalanx. The patient had a visibly enlarged thumb distal phalanx including the nail plate but was neurovascularly intact. The distal phalanx was tender to palpation. He was able to flex and extend the thumb interphalangeal joint approximately 30 degrees each.

Imaging and pathology

**Figure 1:**
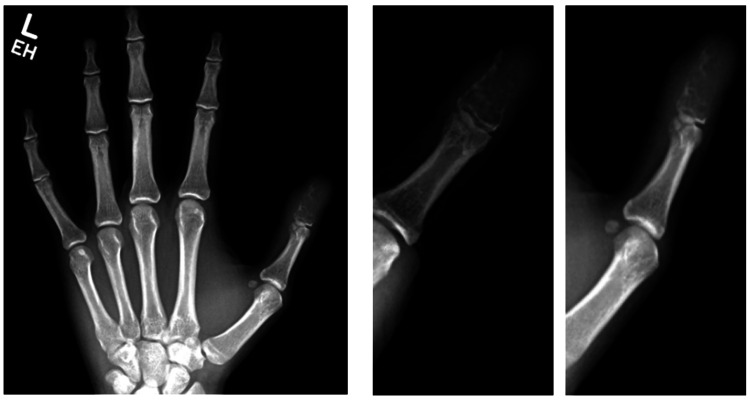
Preoperative X-rays of the left hand and thumb Images show an expansile, lytic lesion of the left thumb distal phalanx with cortical erosion.

X-rays showed an expansile lytic lesion and near-complete erosion of the distal phalanx (Figure 1).

**Figure 2:**
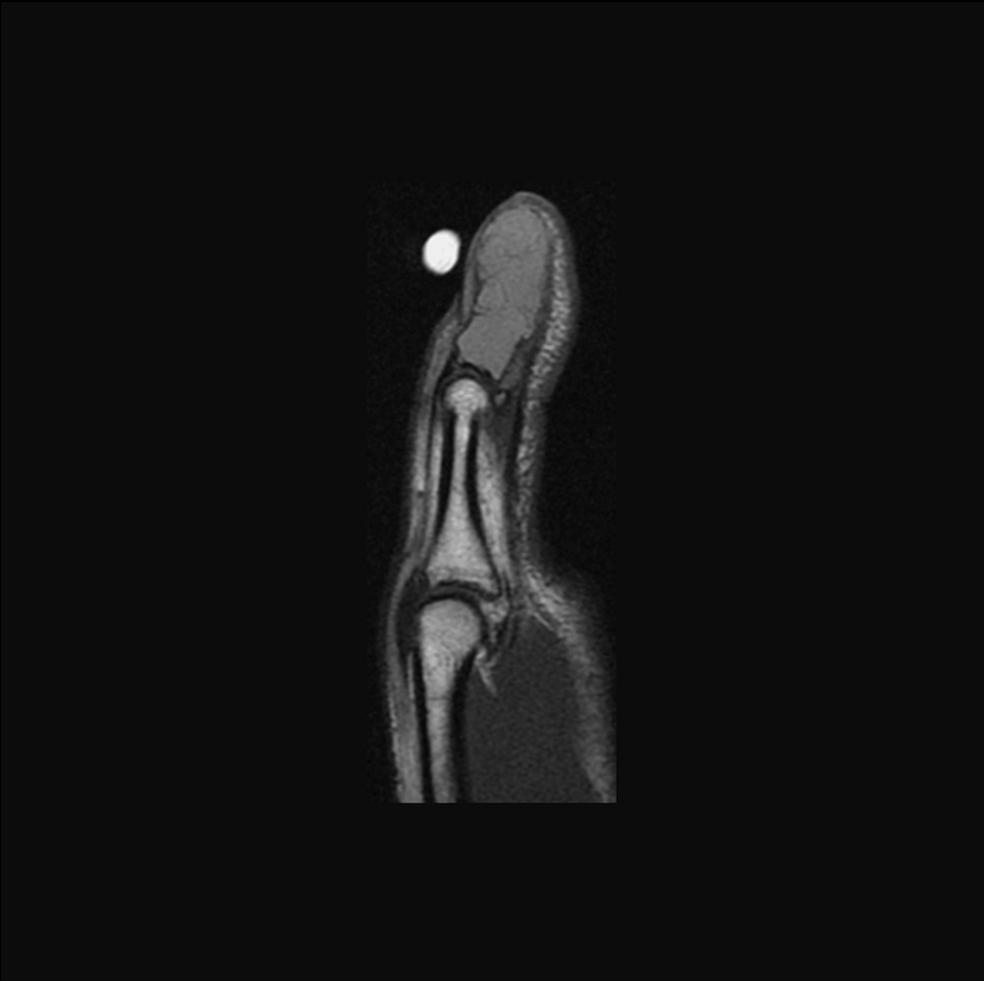
Preoperative sagittal T1 MRI of the left thumb The image is showing an expansile, multiloculated, cystic lesion of the thumb distal phalanx.

MRI was consistent with an aneurysmal bone cyst with intact insertions of the flexor and extensor tendons to the remaining thin cortex (Figure 2).

**Figure 3:**
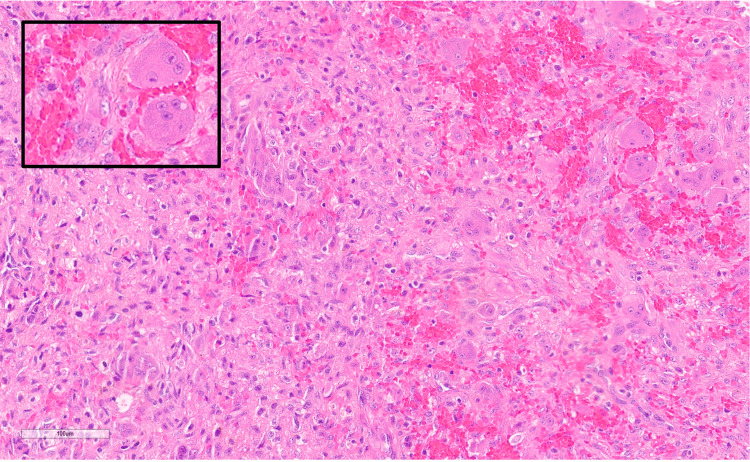
Microscopic appearance of the lesion Hematoxylin and eosin stain, 200X magnification The image is showing fibroblastic stroma and multiple giant cells predominantly around the areas of hemorrhage. Inlay: magnification of giant cells adjacent to hemorrhage. Occasional hemosiderin deposits are also seen.

Initially, the patient underwent a diagnostic open biopsy. The palmar cortex of the distal phalanx was entered through a midline longitudinal palmar incision, and the intramedullary contents were sent for culture and biopsy. Pathology revealed spindle-shaped fibroblasts and collagenous stroma with multiple giant cells around areas of hemorrhage and focal osteoid formation, consistent with a GCRG with a secondary aneurysmal component (Figure 3). Cultures were negative. Serologies for calcium, magnesium, and phosphorous were within normal limits.

Surgery

**Figure 4:**
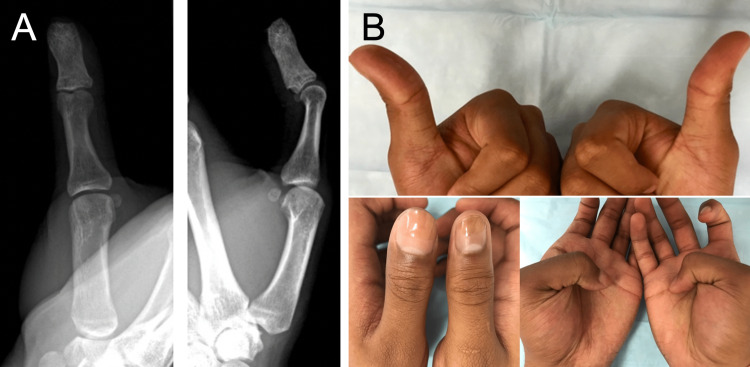
X-rays and clinical pictures of the left thumb seven months postoperatively X-ray images (A) and clinical pictures (B) are showing the formation of the distal phalanx in an appropriate position in relation to the proximal phalanx with an intact range of motion.

At the time of definitive surgical intervention three weeks later, 1.5cc of compacted cancellous autograft was harvested from the ipsilateral distal radius first (to avoid cross-contamination) and then combined with an equal amount of allograft cancellous bone graft. The autograft donor site was then filled with allograft cancellous bone chips. The previous midline palmar incision was then re-incised over the thumb distal phalanx. It was noted that there was only an extremely thin palmar cortical shell remaining. The entire cavity was curetted and irrigated, carefully preserving the flexor pollicis longus (FPL) and extensor pollicis longus (EPL) tendinous attachments. The bone graft was then carefully packed into the defect and confirmed by fluoroscopy that the entire cavity had been completely filled with bone graft. Postoperatively, the patient was placed into a non-removable thumb spica splint for four weeks, when motion was allowed. The full activity was permitted at three months postoperatively when radiographic healing was complete (Figure 4).

Follow Up

One year postoperatively, the patient had thumb interphalangeal joint flexion to 45 degrees and hyperextension of 20 degrees (Figure 4). The thumb interphalangeal joint was completely stable. He continues to be neurovascularly intact and able to perform activities of daily living without any limitations, including contact sports.

## Discussion

GCRGs were first described by Jaffe in 1953 and were thought to be limited to the mandible and maxilla, though there have been multiple case reports describing GCRG in other areas of the body, mainly in the bones of the hand and foot [1-8]. To our knowledge, there has not been a detailed description of treatment of a GCRG involving the distal phalanx of the thumb.

GCRGs are benign tumors causing expansile, lytic lesions of bone usually without evidence of cortical destruction, periosteal reaction, or soft tissue extension [9]. Presenting symptoms may include pain and swelling at the tumor site that may be attributed by the patient to prior trauma [4]. The presentation and characteristics of GCRG are nonspecific and the differential diagnosis includes aneurysmal bone cyst, giant cell tumor of bone, and brown tumor of hyperparathyroidism [2]. Serologies, including serum calcium, phosphorous and alkaline phosphatase, are helpful in ruling out brown tumors of hyperparathyroidism [3]. A confirmatory biopsy is prudent prior to definitive surgical intervention. Histologically, these lesions show a fibrous stroma with spindle-shaped fibroblasts, as well as giant cells [2,9]. Unlike giant cell tumors of bone, the giant cells in GCRG tend to aggregate around areas of hemorrhage [5]. Having a definitive diagnosis of GCRG is important as giant cell tumors of bone can metastasize in rare instances.

The treatment of GCRG is often curettage and bone grafting for primary cases, or amputation/excision for extremely destructive lesions, and in cases of recurrence. Curettage and bone grafting can be successful given that these lesions usually do not completely erode the cortical bone. As demonstrated by Wold et al, GCRG treated with curettage has a higher rate of local recurrence, though can allow for complete functionality in certain cases [4]. However, in their case series, over 50% of recurrences occurred prior to one year postoperatively. Case reports of successful functional outcomes following ray resection for both primary and recurrent GCRG demonstrate its utility in treating these lesions [6,10]. 

Additionally, there have been case reports describing other reconstructive surgical interventions for GCRG lesions. Ugwonali et al. describe the case of a pediatric patient with a recurrent GCRG of the third metacarpal treated with metacarpal excision and fibular autografting [7]. Macdonald et al. describe a case of a patient with GCRG who underwent en bloc resection and reconstruction using iliac crest bone graft and fascial arthroplasty [8]. Finally, Monacelli et al. described a case in which a patient with separate GCRG lesions of the third finger proximal and middle phalanges underwent resection and reconstruction with hydroxyapatite graft and wire stabilization [5]. All of these patients had acceptable functional outcomes.

We believe that this is the first case report specifically discussing the nuances of treating this lesion in the thumb distal phalanx. The limitation of this case report is the limited follow-up postoperatively, though over half of the patients in the Wold et al. series experienced recurrence prior to one year postoperatively [4]. Despite near-complete expansion/obliteration of the entire cortex of the distal phalanx, curettage and bone grafting of the cavity allowed for eventual healing of the bone, and preservation of the soft tissue tendinous insertions such that excellent interphalangeal joint motion was retained. This case report demonstrates that thorough curettage and careful packing of the defect with auto and allograft can result in successful treatment of this lesion.

## Conclusions

Our patient had a GCRG of the thumb distal phalanx, which presents different functional concerns than lesions elsewhere in the hand. This is the first case in the literature to specifically discuss the nuances involved with treating a lesion here. Despite having almost no cortical bone left surrounding the lesion, this patient underwent curettage and bone grafting, which allowed for the best functional outcome, given the intact soft tissue attachments. The patient has continued to do well and has no functional deficits.
